# Evaluation of the microbial growth response to inorganic nanoparticles

**DOI:** 10.1186/1477-3155-4-3

**Published:** 2006-02-28

**Authors:** Darryl N Williams, Sheryl H Ehrman, Tracey R Pulliam Holoman

**Affiliations:** 1 Department of Chemical and Biomolecular Engineering, University of Maryland, 2113 Building 090, College Park, MD 20742, USA

## Abstract

In order to enhance the utilization of inorganic nanoparticles in biological systems, it is important to develop a fundamental understanding of the influence they have on cellular health and function. Experiments were conducted to test silica, silica/iron oxide, and gold nanoparticles for their effects on the growth and activity of *Escherichia coli *(*E. coli*). Transmission electron microscopy (TEM) and dynamic light scattering (DLS) were used to characterize the morphology and quantify size distribution of the nanoparticles, respectively. TEM was also used to verify the interactions between composite iron oxide nanoparticles and *E. coli*. The results from DLS indicated that the inorganic nanoparticles formed small aggregates in the growth media. Growth studies measured the influence of the nanoparticles on cell proliferation at various concentrations, showing that the growth of *E. coli *in media containing the nanoparticles indicated no overt signs of toxicity.

## Background

Research concerning the impact of inorganic nanoparticles on cellular health will enable new developments in nanobiotechnology to reach their fullest potential. An improved understanding of nanoparticles and biological cell interactions can lead to the development of new sensing, diagnostic, and treatment capabilities, such as improved targeted drug delivery, gene therapy, magnetic resonance imaging (MRI) contrast agents, and biological warfare agent detection [1-6]. What is not certain about the production of these particles is whether they, alone, are toxic to cells in general.

Cytotoxicity is of major concern and will become increasingly so as the demand for nanoparticles grows with the development of more biological applications. Questions, such as how and if nanoparticles harm biological environments, how persistent they may be, and to what degree they affect other organisms including people are all concerns. It is known that nanoparticles can transfect cells; however, responses to nanoparticles inside and outside of cells are unknown. As nanoparticles become more common and widely produced, the chances of unplanned events leading to their dissemination and accumulation in the environment increase, and could lead to unforeseen changes to biological systems. In this study, *E. coli *served as a representation of how cells might respond to the presence of nanoparticles in their growth environment.

The goal of the research presented here is to investigate how nanoparticles interact with microbial cells, and what effect nanoparticles have on their growth process. Nanoparticles present a research challenge because little is known about how they behave in relation to microorganisms, particularly at the cellular level. The colloidal behavior of the inorganic nanoparticles in the microbial growth media was investigated to determine the stability of these systems in saline environments. Colloidal stability is an issue when dealing with biological environments due, in part, to the effect of salt on the nanoparticles. Agglomeration occurs causing sedimentation of the nanoparticles, thus limiting their interactions with growing cells, such as *E. coli*.

Three types of nanoparticles were used to conduct this study: silica, silica/iron oxide, and gold. The silica/iron oxide nanoparticles are important because of their magnetic properties. They could potentially be used for medical applications, such as MRI and targeted drug delivery applications. Another application would be to use them as biological sensors. Being that they are composites, the silica portion of the nanoparticle can be functionalized to attract various biological elements while the iron oxide portion can provide mobility under the presence of a magnetic field.

Gold nanoparticles are employed in multiple applications involving biological systems. Gold has exceptional binding properties, and this makes it attractive for attaching ligands to enhance various biomolecular interactions. These nanoparticles also exhibit an intense color in visible region for spectroscopic detection and also great contrast for electron microscopic imaging [7]. Despite all of these applications for gold nanoparticles, there is still little knowledge as to how these colloid systems effect microbial environments. Silica nanoparticles are favorable because they are inexpensive, easy to produce, and have surface hydroxide groups that make them easy to functionalize

## Results and discussion

### TEM measurements

**Figure 1:**
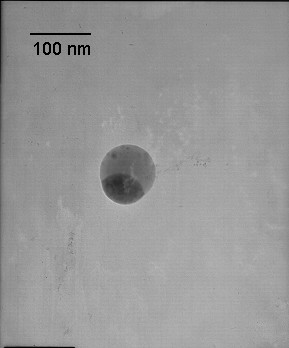
Transmission electron microscope image of a SiO_2_/γ-Fe_2_O_2 _particle generated in a premixed flame.

**Figure 2:**
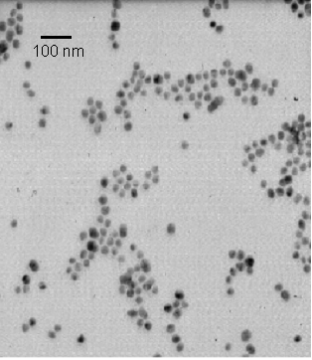
Transmission electron microscope image of gold particles without PEG coating (Majetich).

**Figure 3:**
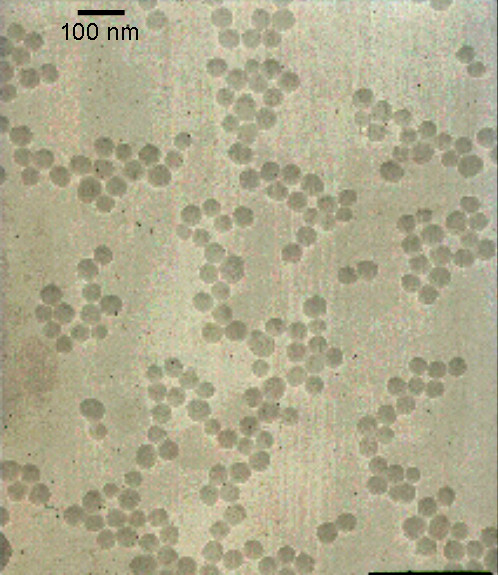
Transmission electron microscope image of silica nanoparticles.

According to the micrographs, the morphology of the silica/iron oxide nanoparticles is approximately spherical. The mole ratio of silicon to iron is roughly 1:1, and Figure 1 shows the nanoparticles with the dark side being iron oxide and the lighter side being silica. The average particle size was 80 nm. Figure 2 is a micrograph of the gold nanoparticles indicating that they are also spherical in shape. Lastly, the silica nanoparticles were analyzed using TEM, and the results show spherical morphology with an average particle size of 60 nm. Figure 3 is a micrograph of the silica nanoparticles taken in aqueous solution.

**Figure 4:**
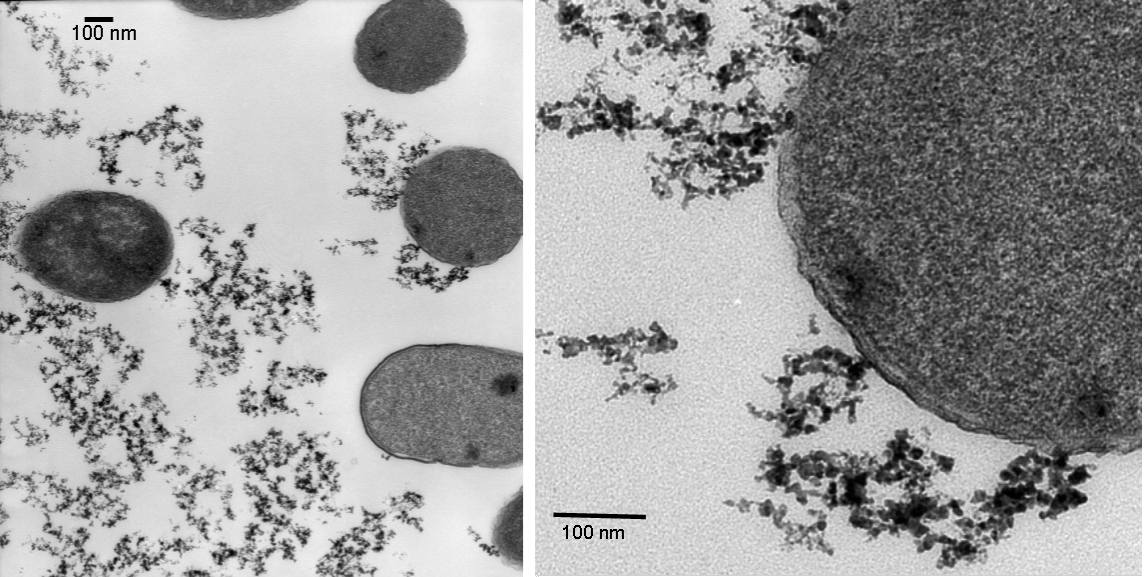
(a) Transmission electron microscope image of cross sections of *E. coli *grown with composite iron oxide nanoparticles. (b) A close up image showing the composite nanoparticles and a cell in contact with each other.

The micrographs of *E. coli, *in the presence of iron oxide composite nanoparticles, indicate that the cells are able to maintain growth, showing no overt signs of toxicity. A mixture of circular and elongated cell cross sections are seen. It should be noted that *E. coli *is characteristically rod-shaped. The cells were sectioned and thus the shape is a function of the angle of each cell relative to the diamond knife during sectioning. TEM images of cells grown in the absence of nanoparticles also showed a similar distribution of cross section shapes. There appears to be an association of the nanoparticles with the cell membrane, as shown in Figures 4a and 4b for *E. coli *and composite iron oxide nanoparticles. Based upon the fact that the surfaces of the nanoparticles are either bare or PEG coated, it is likely that the interaction between the particles and the membrane is non-specific rather than specific between the nanoparticles and a particular component of the membrane such as a surface expressed protein.

### DLS measurements

Nanoparticles have a tendency to agglomerate in solution due, in part, to the characteristics of the liquid medium with the addition of salt. In regards to the nanoparticles and microbial cell interactions, this will greatly affect the behavior of the cells. Non-agglomerated particles suspended in solution are preferable for testing purposes because of the following:

1) free moving, single unit particles have more contact with microbes.

2) translocation through the cell membrane will be accelerated due to size.

LB media contains a high salt concentration (0.2 M) that may contribute to the agglomeration of the nanoparticles. The surface charge on the nanoparticles in solution allows the nanoparticles to attract to one another because of the influence of ions from the salts, therefore resulting in the formation of large agglomerates [8-10]. As a result, the nanoparticles may fall out of solution and settle to the bottom of the shake flasks.

**Figure 5:**
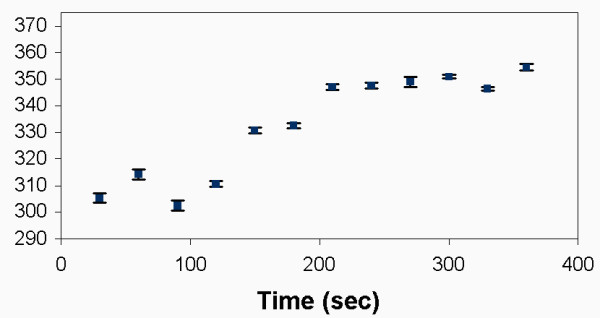
Results of dynamic light scattering measurements for silica in LB media show that the nanoparticles agglomerate in solution.

**Figure 6:**
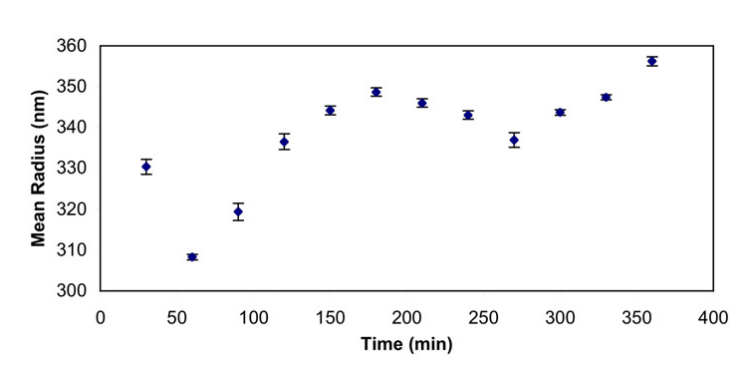
Dynamic light scattering measurement of silica/iron oxide particles in LB media.

**Figure 7:**
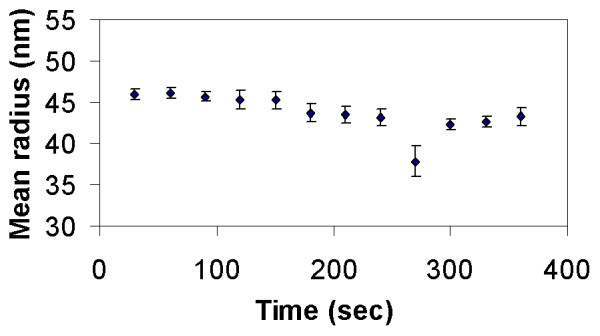
PEG-coated Au nanoparticles show greater stability in LB media than silica and silica/iron oxide composite nanoparticles. PEG-coated Au remains at approximately 46 nm.

**Figure 8:**
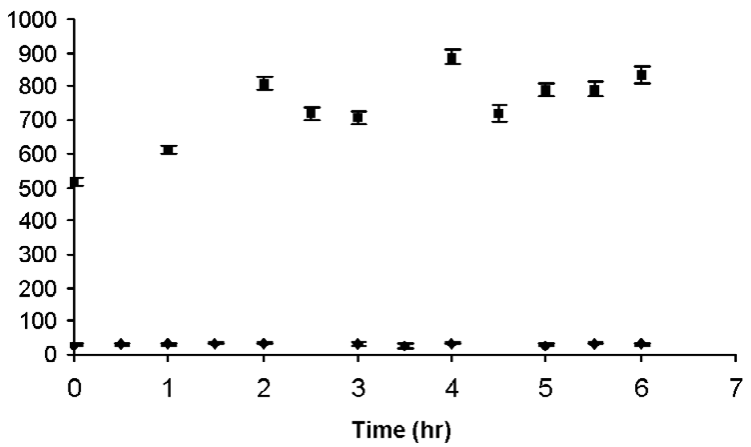
Dynamic light scattering measurements of PEG modified Au nanoparticles (◆) and iron oxide composite nanoparticles (■) measured during a time course of 6 hours in LB media. During this run, the Au particles remained stable and showed little change in hydrodynamic radius. The iron oxide nanoparticles, however, were less stable as indicated by their increasing size.

As time increased, the mean particle radius increased. The agglomerate size for the silica particles ranged between 300–360 nm, as shown in Figure 5. Figure 6 shows DLS measurements for silica/iron oxide, indicating similar behavior in the LB media. In contrast, Figure 7 shows the PEG-coated gold nanoparticles remained stable in LB media, thus retaining their size without forming large agglomerates. DLS measurements were taken over the time frame correlating with the growth measurements to determine how the particles behaved during that process. Figure 8 is a comparison of the Au particles with the composite particles in suspension for a duration of six hours. Again, the Au particles maintain their size and stability.

### Growth experiments

**Figure 9:**
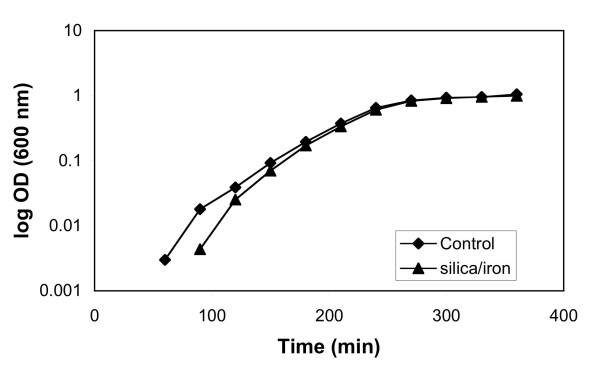
Growth curves of *E. coli *in the presence of 2.2 × 10^-3^g/ml silica/iron oxide nanoparticles.

The overall results indicated that the growth of *E. coli *exposed to silica, silica/iron oxide, and gold nanoparticles was uninhibited. Growth curves were generated for *E. coli *growing in 100 mL of LB media containing silica/iron oxide nanoparticles at a concentration of 2.2 × 10^-3 ^g/mL of solution. Under these growth conditions, there was no evidence that the nanoparticles prevented the microbial cells from growing. Figure 9 illustrates the growth curves for *E. coli *growing with and without silica/iron oxide nanoparticles. Results indicate that there is little difference between the two curves.

**Figure 10:**
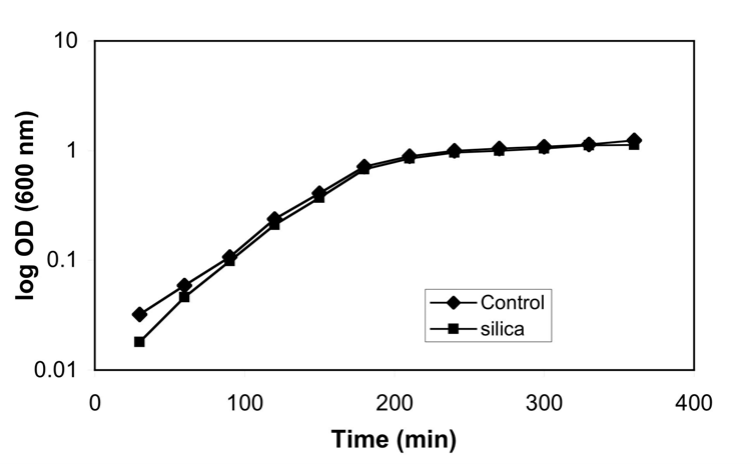
Growth curves for *E. coli *in the presence of 3.3 × 10^-2 ^g/ml silica nanoparticles.

Another experiment was conducted using pure silica nanoparticles at the same volume as the silica/iron oxide experiment. Growth data was taken for 3.3 × 10^-2 ^g/mL of silica solution in 100 mL of LB media. Figure 10 depicts the growth curves for this experiment.

**Figure 11:**
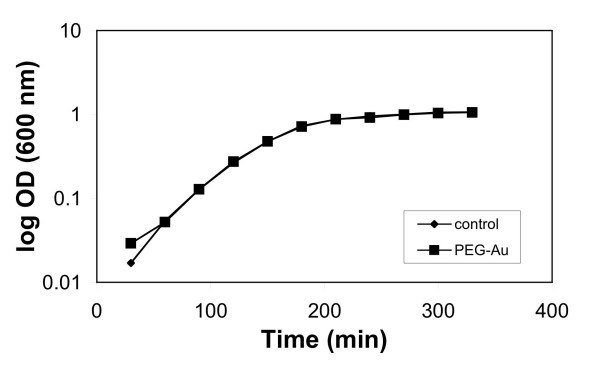
Growth curves of *E. coli *in the presence of 1.1 × 10^-4 ^g/ml PEG-coated gold nanoparticles.

As with the silica and silica/iron oxide experiments, a toxicity study was performed using the gold nanoparticles at a concentration of 1.1 × 10^-4 ^g/mL. The gold particles show greater stability in solution due to the coating of PEG on the surface. This allows the particles to stay suspended in solution; also, they are able to sustain their initial radius of 30 nm. Figure 11 indicates that there is little, if any, effect on growth resulting from the presence of the gold nanoparticles.

**Figure 12:**
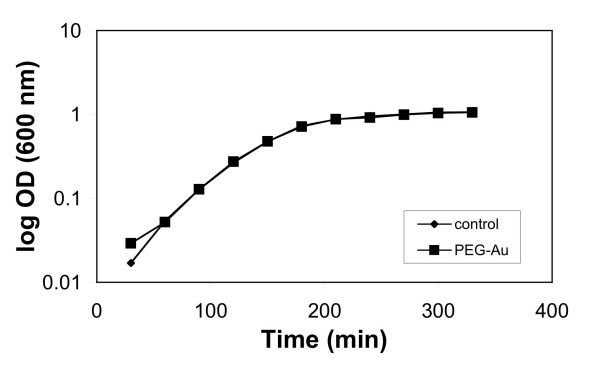
Growth curves of *E. coli *in the presence of higher concentrations of composite iron oxide nanoparticles.

A final experiment was conducted to test the influence of the composite iron oxide nanoparticles on *E. coli *at concentrations much higher than the previous experiments. The amount of the composite nanoparticles was increased to 2.2 × 10^-2 ^g/mL in one flask and 4.4 × 10^-2 ^g/mL in a separate flask. Optical density measurements, given in Figure 12, showed that *E. coli *was not inhibited by the increase in nanoparticle concentration.

### Magnetic experiment

**Figure 13:**
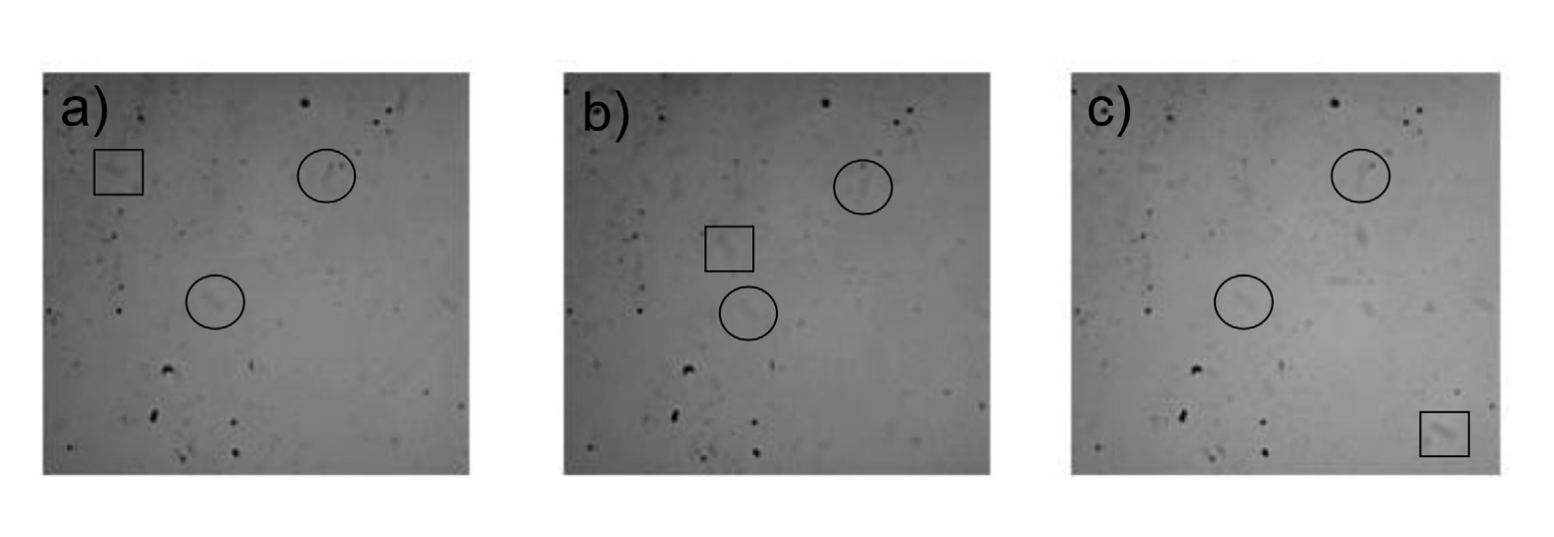
Three stills showing the movement of *E. coli *under the presence of a magnetic field. These cells, placed on a glass slide, were taken from a cell suspension growing with 2.2 × 10^-3 ^g/ml silica-iron oxide nanoparticles. A cylindrical neodymium-iron-boron permanent magnet (Arbor Scientific, Ann Arbor, MI) was placed two inches away on the right hand side of a glass slide sitting on a digital confocal microscope for imaging. Some cells exhibited motion along the magnetic field lines, but not all of the cells moved in the direction of the field suggesting that not all cells were in contact with nanoparticles. Image (A) shows a cell (square box) moving from the upper left hand corner between two stationary cells (circled) to the lower right hand corner (C) which is the direction of the magnetic pull on the particles.

A 100 ml suspension of cells placed on a glass slide were taken from a cell suspension growing with 2.2 × 10^-3 ^g/ml silica-iron oxide nanoparticles. A cylindrical neodymium-iron-boron permanent magnet (Arbor Scientific, Ann Arbor, MI) was placed two inches away on the right hand side of a glass slide sitting on a digital confocal microscope for imaging. Figure 13 depicts the movement of *E. coli *as a result of the external magnet positioned next to the slide. Some cells exhibited motion along the magnetic field lines (indicated by the square in Figure 13), but not all of the cells moved in the direction of the field (cells highlighted with circles). This suggests that not all cells were in contact with nanoparticles, and also that the cells were not being swept along by bulk fluid motion resulting from motion of magnetic nanoparticles alone.

## Conclusion

Preliminary studies were performed to determine if nanoparticles affect the growth of microbial cells by studying cell cultures in the presence of several inorganic nanoparticles. Experimental evidence indicated that the interactions between *E. coli *and the nanoparticles used during this study were nonspecific. *E. coli *showed no overt signs of growth inhibition using the methods presented in this paper. Of course, it is possible that there may be more subtle changes in cell function and behavior detectable at the gene or protein level. For the purpose of this study, it was important to show preliminary results that describe the effects of inorganic nanoparticles under normal growth conditions using known methods for measuring microbial cellular growth. However, as a cautionary note, the results presented are not meant to be generalized beyond the material and biological systems and conditions reported here.

## Methods

### Inorganic nanoparticles

Table I gives the specifications for each type of nanoparticle (silica, silica/iron oxide, and gold) used in this study. The silica nanoparticles were made by base catalyzed hydrolysis of TEOS [11]. Silica/iron oxide nanoparticles were flame-generated from iron pentacarbonyl and hexamethyldisiloxane in a premixed methane/oxygen/nitrogen flame [12]. Each particle contains both gamma-Fe_2_O_3 _and silica in a 1:1 molar ratio. Lastly, the gold particles were produced via sodium citrate reaction with HAuCl_4 _in water followed by the addition of polyethylene glycol (PEG) to coat the surface [13].

**Table 1:** Characteristics of the inorganic nanoparticles used in experimentation.

	Mean radius (nm)	Concentration per flask (g/mL)	Crystalline structure	Surface chemistry
Silica	60 ± 1.3	3.3 × 10^-2^	amorphous	hydrophilic
Silica/Iron Oxide	80 ± 2.5	2.2 × 10^-3^	amorphous silica, crystalline iron oxide	hydrophilic
PEG-coated Au	30 ± 0.15	1.1 × 10^-4^	crystalline	hydrophilic

### Culture media and culture conditions

For rapid growth of the microbial cells, Luria Bertani (LB) medium was prepared and sterilized for each experiment. A set of 250 mL shake flasks were also sterilized before experimentation. 100 mL of LB medium was transferred to each flask. Various concentrations of nanoparticles were carefully placed into each flask, leaving one as a control to track the normal growth of the microbial cells without nanoparticles. Experiments were performed using both a negative control (flask containing cells plus media) and a positive control (flask containing nanoparticles plus media). Both of the negative and positive control values obtained from optical density measurements were subtracted from the experimental values (flasks containing cells, media, and nanoparticles). The growth curves represent the difference between the controls and the experimental values.

Each flask was then inoculated with 1 mL of *E. coli *(pBR322 JM105) grown in liquid LB medium. The flasks were shaken at 180 rpm and 37°C in a shaking water bath. Optical density measurements from each flask were taken every thirty minutes to record the growth of the microbes from inoculation through late exponential phase using a spectrophotometer set at 600 nm. The growth rate of microbial cells interacting with the nanoparticles was determined from a plot of the log of the optical density versus time.

### Particle morphology using transmission electron microscopy

Transmission electron microscopy (TEM) was used to obtain images of the nanoparticles. Silica/iron oxide samples were prepared for TEM imaging by inserting a TEM grid (copper coated with formvar) into dry powder using tweezers to hold the grid. The sample grid was then lightly tapped to remove any excess particles, and the grid was placed in the TEM for imaging. The silica and gold nanoparticles were in suspension, and samples were prepared by inserting the TEM grid into each liquid sample. The sample grids were then allowed to air dry overnight.

### Characterization of nanoparticles by dynamic light scattering

One of the more common methods employed to characterize pharmaceutical colloids is dynamic light scattering (DLS). Analysis of the size distribution of the nanoparticles was performed using a DLS autocorrelation tool known as Photocor^®^. DLS measurements were taken of the nanoparticles in distilled water and in LB growth media. With this procedure, the difference between the behavior of the nanoparticles in solutions with and without salt was compared.

### Nanoparticle/cell interaction studies using TEM

After characterizing the various nanoparticles, experiments were conducted to observe the relationship between the iron oxide composite nanoparticles and *E. coli *in LB media. Cell/nanoparticle interactions were observed using a Zeiss EM10 CA transmission electron microscope at the University of Maryland Biological Ultrastructure Facility. Samples of *E. coli *were withdrawn at points during late exponential phase (optical density ~0.6). After collection, they were centrifuged and suspended at room temperature in 0.12 M Millonig's phosphate buffer at pH 7.3 and later with 2% glutaraldehyde. The cell pellets were then washed again with buffer, and then secondary fixed with 1% OsO_4_. At this point, they were washed with distilled water and then postfixed with 2% uranyl acetate, rinsed in buffer and double distilled water, dehydrated in a series of ethanol and propylene oxide immersions, and embedded in Spurr's resin. A diamond knife was used to section the embedded cells. The sections were post-stained with 2.5% aqueous uranyl acetate and 0.2% aqueous lead citrate.

## Competing interests

The author(s) declare that they have no competing interests.

## Authors' contributions

DW carried out all experimentation and data analysis. SH and TH conceived of the study and participated in its design and coordination and helped to draft the manuscript. All authors read and approved the final manuscript.
